# Case Report: Toripalimab: a novel immune checkpoint inhibitor in advanced nasopharyngeal carcinoma and severe immune-related colitis

**DOI:** 10.3389/fimmu.2023.1298902

**Published:** 2023-11-24

**Authors:** Canhua Luo, Huangwei Chen, Huihuan Wu, Yongjia Liu, Guoyin Li, Weijian Lun

**Affiliations:** Department of Gastroenterology, The Sixth Affiliated Hospital, School of Medicine, South China University of Technology, Foshan, Guangdong, China

**Keywords:** Toripalimab, nasopharyngeal carcinoma, autoimmune colitis, immune-related adverse event, CMV infection

## Abstract

Toripalimab, a specific immune checkpoint inhibitor targeting the programmed death 1 (PD-1) receptor, represents a novel immunotherapeutic approach for advanced nasopharyngeal carcinoma, showing promising curative potential. However, it is not without drawbacks, as some patients experience immune-related adverse events (irAEs) associated with this treatment, and there remains a limited body of related research. Here, we present a case of advanced nasopharyngeal carcinoma in a patient who developed colitis as an irAE attributed to Toripalimab. Subsequent to Toripalimab treatment, the patient achieved complete remission. Notably, the development of colitis was accompanied by inflammatory manifestations evident in colonoscopy and pathology results. Further investigation revealed cytomegalovirus (CMV) infection, detected through immunohistochemistry in 11 colon biopsies. Subsequent treatment with ganciclovir and steroids resulted in symptom relief, and colonoscopy indicated mucosal healing. Our case highlights the association between irColitis induced by Toripalimab and CMV infection. Toripalimab demonstrates remarkable efficacy in treating advanced nasopharyngeal carcinoma, albeit with a notable risk of irAEs, particularly in the form of colitis. The link between symptoms and endoscopic pathology findings in irColitis is noteworthy. Standardized biopsy procedures can effectively confirm the diagnosis of CMV infection. Our findings may provide valuable guidance for managing acute CMV infection and irAEs associated with Toripalimab in the treatment of nasopharyngeal carcinoma in the future.

## 1 Introduction

Targeting the programmed cell death protein 1 (PD-1) or its ligand (PD-L1) immune checkpoint pathway has demonstrated its effectiveness as a therapeutic strategy for malignant tumors (1). However, the use of immunotherapy in cancer treatment can lead to a wide variety of immunotherapy-related adverse events (irAEs), which can range from mild to severe. The most common adverse effects of this class of drugs are gastrointestinal in nature, occurring in approximately 35% of cases for CTLA-4 inhibitors and approximately 20% for PD-1 inhibitors (2).

In some cases, patients may even experience opportunistic infections, such as cytomegalovirus (CMV) infection (3) and *Nocardiosis* infection (4). Nasopharyngeal carcinoma (NPC) is a highly invasive and metastatic cancer, and unfortunately, there are no FDA-approved treatments available for NPC patients (5).

**Figure 1:**
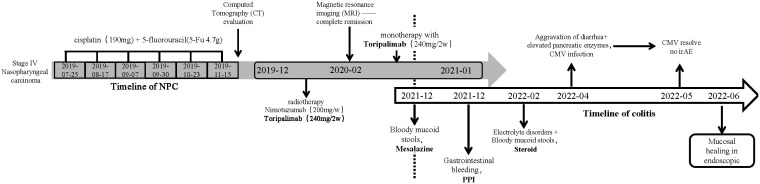
Timeline of events.

Toripalimab, a PD-1 inhibitor, has been granted breakthrough designation and orphan drug designations by the FDA for the treatment of NPC. It holds great promise as the first immunotherapy for NPC treatment in the USA. This report describes the progression of multiple adverse effects, including gastrointestinal issues and CMV infection (such as elevated pancreatic amylase, diarrhea/colitis, and electrolyte disturbances), in a patient with multiple metastatic nasopharyngeal carcinoma undergoing treatment with Toripalimab (**Figure 1**). This case highlights the challenges and complexities associated with immunotherapy in the context of NPC treatment.

## 2 Case presentation

A 62-year-old man with no history of inflammatory bowel disease was diagnosed with advanced NPC in May 2019. The pathological diagnosis revealed non-keratinizing undifferentiated carcinoma. Immunohistochemistry results showed cytokeratin (CK) positivity (CK+), EGFR (EP22) positivity [EGFR (EP22+)], p63 positivity (focally) (p63+), and positive results for EBER *in situ* hybridization (EBER+). A positron emission computed tomography (PET/CT) scan indicated multiple small lung and liver nodules and increased bone metabolism in the L1–L2 vertebrae, suggestive of lung, liver, and vertebral metastases.

Starting in July 2019, the patient underwent front-line chemotherapy with a combination of cisplatin (190 mg) and 5-fluorouracil (5-Fu, 4.7 g), with a total of six treatment cycles. The first evaluation, conducted by computed tomography (CT) scan at the 18th week of treatment, revealed a reduction in the size of the nasopharyngeal carcinoma and lung and liver metastases, indicating partial remission. However, the bone metabolism in the L2 vertebral body remained stable, leading to a stable disease (SD) evaluation.

From December 2019 onwards, the patient received a combination treatment regimen consisting of radiation therapy (PGTVnx DT70Gy/33F, PGTVnd DT68Gy/33F, PTV1 DT62Gy/33F, and PTV2 DT54Gy/33F), nituzumab (200 mg/week), and toliperizumab (240 mg every 2 weeks). Subsequent magnetic resonance imaging (MRI) scans in February 2020 demonstrated resolution of the nasopharyngeal tumor, cervical lymph nodes, and liver metastases, indicating complete remission. The bone metabolism in the L1 and L2 vertebrae also showed a decrease. Currently, the patient continues maintenance therapy with Toripalimab as a monotherapy, administered every 2 weeks.

In December 2021, the patient developed diarrhea with watery and mucus stools, experiencing almost four to six episodes per day. Oral mesalazine treatment was initiated, which converted the watery stools into loose stools, occurring one to two times per day. In January 2022, the patient presented with melena, which was suspected to be due to upper gastrointestinal bleeding. The patient was discharged after receiving proton pump inhibitors (PPIs) treatment.

**Figure 2:**
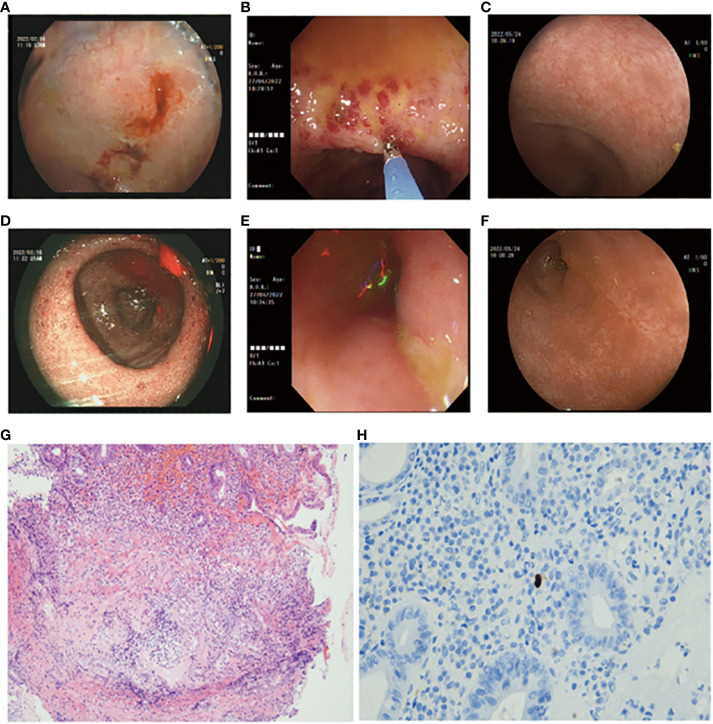
**(A, D)** Colonoscopy images depicting erythema, erosions, and granular changes in the ileocecal region (February, 2022). **(B, E)** Colonoscopy images showing multiple erosions, erythema, and ulcers of the colonic mucosa (April, 2022). **(C, F)** Colonoscopy images displaying endoscopic healing of the colonic mucosa after treatment (May, 2022). **(G)** Hematoxylin and Eosin (H&E)-stained section of the colonic biopsy indicating diffuse colitis with crypt atrophy, loss of crypts, and inflamed lamina propria. Lymphoplasmacytic infiltration of the lamina propria with increased intraepithelial lymphocytes and crypt epithelial cell apoptosis is evident. **(H)** Immunohistochemical staining revealing cells positive for anti-CMV antibodies (arrows).

However, the diarrhea with watery and bloody stools recurred, along with electrolyte disturbances in February 2022 (**Figures 2A, D**). A colonoscopy revealed granular changes in the ileocecal region. Symptoms improved after treatment with mesalazine and steroids, but the patient discontinued the drugs on his own during follow-up.

In April 2022, the patient experienced a worsening of symptoms, including the presence of watery diarrhea up to 10 times a day and a weight loss of nearly 4 kg. Following admission to the hospital, a colonoscopy was conducted, revealing multiple areas of ulceration, erythema, and mucosal erosion in the colon, accompanied by elevated levels of amylase, lipase, and electrolyte imbalances (**Figures 2B, E**). Immunohistochemical analysis of colon biopsy specimens confirmed the presence of CMV. The patient received a 2-week course of intravenous ganciclovir treatment (0.25 g bid day). After the treatment, the consistency of the stool changed from bloody to loose and yellow, with a frequency of three to five times per day. Subsequently, we initiated a re-treatment with methylprednisolone tablets, a corticosteroid. After 1 week of treatment, the levels of amylase and lipase improved, and the stool became formed, with a frequency of approximately one to two times per day. A follow-up colonoscopy in May 2022 revealed healing of the colonic mucosa, confirming the success of the treatment (**Figures 2C, F**).

## 3 Discussion

PD-1/PD-L1 immune checkpoint inhibitors have received approval for the treatment of various advanced cancers, including melanoma, kidney cancer, head and neck cancer, and non-small cell lung cancer (6). However, there is currently no approval for the use of these inhibitors in the treatment of nasopharyngeal cancer in either the European Union or the United States.

Toripalimab, a PD-1 inhibitor developed by Shanghai Junshi Biosciences, binds primarily to the FG loop of PD-1 with an unusually long complementarity-determining region 3 loop of the heavy chain. This binding pattern is distinct from the known epitopes of anti-PD-1 monoclonal antibodies and is supported by structural evidence (7).

A multicenter randomized phase 3 trial of Toripalimab or placebo in combination with chemotherapy as a first-line treatment for advanced nasopharyngeal cancer demonstrated a significant improvement in progression-free survival (PFS) in the Toripalimab arm. At the prespecified interim PFS analysis, the median PFS in the Toripalimab arm was 11.7 months compared to 8.0 months in the placebo arm, with a hazard ratio (HR) of 0.52 (95% confidence interval (CI), 0.36–0.74) and a p-value of 0.0003. This improvement in PFS was consistent across key subgroups, including those with PD-L1 expression [HR = 0.603 (95% CI, 0.364–0.997)] (8). In China, Toripalimab has been approved for the treatment of nasopharyngeal carcinoma.

In this case, the patient was diagnosed with stage IV nasopharyngeal carcinoma with bone and liver metastasis. The initial chemotherapy regimen of 5-Fu and cisplatin resulted in a partial response (PR). Subsequently, Nimotuzumab and Toripalimab, in combination with radiotherapy, were used as a second course of treatment. In line with the findings of the phase 3 trial, re-examination after chemotherapy showed regression of the nasopharyngeal malignancy and cervical lymphadenopathy.

However, during the subsequent maintenance treatment, the patient developed grade 2 diarrhea, along with four to six episodes of vomiting and bloody stools per day. Steroid therapy provided initial relief, but the symptoms worsened to 10-15 watery stools per day. Gastrointestinal toxicity represents the most severe and common immune-related adverse event (irAE) associated with the increased use of immune checkpoint inhibitors (ICPIs). ICPI-induced diarrhea occurs in up to 30% of patients in clinical trials (9).

Identification of CMV infection is one of the key treatment points in this case, as previous research has shown an association between CMV infection and immune system dysregulation. For instance, studies have indicated that reactivation of cytomegalovirus is more likely to occur in patients receiving corticosteroid therapy (10). Additionally, concomitant use of cyclosporine can lead to a higher rate of CMV reactivation (11). In the case of CMV infection in PD-1-related colitis, the European Society of Clinical Microbiology and Infectious Diseases (ESCMID) has issued a consensus statement, suggesting that immune checkpoint inhibitors (ICIs) inherently do not increase the risk of infection, but immunosuppressive therapies used to manage irAEs can increase the likelihood of opportunistic infections (12). Previous case reports have also emphasized instances of opportunistic infections following immune suppression, including aspergillosis (13), *Pneumocystis jirovecii* pneumonia (PJP) (14), and campylobacteriosis (14). These findings indicate that the primary risk of infection post-ICI treatment may be due to immunosuppression. However, our team considers that in addition to immunosuppression-induced infections, there may also be a possibility of immune dysregulation underlying these infections. This concept, referred to as immunotherapy infections due to dysregulated immunity (ITI-DI), was proposed by Tommaso Morelli (15).

First, as a herpes virus, CMV infects most adults after they reach adulthood and remains latent for a period of time (16). In some reported cases, immunosuppression may have contributed to viral pathogenesis, but pro-inflammatory conditions can do the same thing. As an example, colonic inflammation in inflammatory bowel disease can impair the function of natural killer cells, which can promote viral reactivation in combination with immune-mediated mucosal damage (17). There is evidence that inflammation may play a role in some CMV infections associated with ICIs. ICIs can enhance virus-specific T-cell activity, and subsequent inflammation driven by interleukin (IL)-6 and IL-17 may protect virus-infected cells from apoptosis by expressing Bcl-2 and Bcl-xL, thereby promoting viral persistence (18). Additionally, CTLA-4/PD-1 blockade in monkeys reactivates the simian immunodeficiency virus (SIV) (19). In the context of this case, the patient initially received PD-1-related ICI therapy, followed by subsequent treatment with corticosteroids, thereby increasing the likelihood of CMV infection. A previous research conducted by Dr. McCurdy demonstrated that cytomegalovirus can exhibit patchy distribution in the colon, even in individuals with severe disease (17). Therefore, in ulcerative colitis (UC) and Crohn’s disease (CD) patients, colon biopsies were performed in 11 and 16 cases, respectively, with a positivity rate of 80% (17). In this case, CMV infection was confirmed through 11 biopsies and immunohistochemical analysis (**Figure 2H**). Colonoscopy also revealed colonoscopic features of CMV infection, such as patchy ulceration and linear ulcers (**Figure 2E**). The subsequent treatment with ganciclovir and steroids led to a significant improvement in the patient’s symptoms, as evidenced by follow-up colonoscopy demonstrating endoscopic healing (**Figures 2C, F**).

Although the European Crohn’s and Colitis Organisation’s guidelines on opportunistic infections do not recommend routine CMV screening in IBD patients before initiating immunomodulatory therapy, they suggest excluding CMV infection when patients experience acute steroid-refractory colitis before escalating immunomodulatory therapy (20). However, based on this case, we propose that for all patients who develop PD-1-related colitis, it is strongly advised to conduct comprehensive screening for fecal bacterial cultures, fungi, and relevant viruses such as CMV and actively exclude associated infections. If feasible, high-quality colonoscopy examination and standardized biopsies should be performed. Instead of solely attributing the cause of patients’ colitis to irAE, particularly when treatment with steroids yields poor response or worsening symptoms, it is crucial to actively consider the possibility of an infectious etiology. This is because blindly escalating immunomodulatory therapy may exacerbate pre-existing infection or trigger new immunotherapy infections due to immunosuppression (ITI-IS).

It is worth noting that the endoscopic findings, pathological results, and patient symptoms in this case are consistent. Chen’s study classified PD-1-related colitis into collagenous colitis and lymphocytic colitis, with the most common pathological features being neutrophilic cryptitis, prominent crypt apoptosis, and crypt atrophy/dropout (21). However, Gallo’s study suggests that patients with collagenous colitis do not exhibit macroscopic findings during endoscopic examination (22). Wang’s research found that most patients have persistent endoscopic and histological inflammation regardless of clinical recurrence of diarrhea (23). In the present case, the initial PD-1-related gastrointestinal toxicity resulted in granular changes in the ileocecal region during colonoscopy, with histopathological features including neutrophil aggregation, crypt apoptosis, and crypt abscess (**Figure 2G**). However, these features are not unique, and according to the NCCN guideline for the management of immunotherapy-related toxicities (24), there is no definitive gold standard for diagnosing PD-1-related colitis. Therefore, our team utilized a “timeline comparison” method (**Figure 1**), which involves comparing the patient’s entire chemotherapy timeline with the timeline of onset of colitis symptoms to assess whether there is a temporal correlation. Additionally, we compared the frequency and characteristics of bowel episodes to assess the severity of the condition and aid in identifying potential infections. This approach may serve as a reference for future diagnosis of PD-1-related colitis.

In summary, this case primarily highlights three key points. First, Toripalimab, the latest chemotherapy regimen for advanced nasopharyngeal carcinoma, demonstrates satisfactory treatment efficacy. However, despite its unique binding sites, Toripalimab still carries the risk of inducing related irAEs, consistent with previous literature reports. Second, although the endoscopic and pathological findings of PD-1-related colitis closely correlate with the patient’s symptoms, they currently cannot serve as the definitive diagnostic gold standard. Hence, it is recommended to assist in the diagnosis by comparing the timeline of immunotherapy administration and the timeline of colitis onset. Lastly, as an opportunistic viral infection, both PD-1-induced immunotherapy infections due to dysregulated immunity (ITI-DI) and immunosuppressive therapy employed to treat irAEs caused by opportunistic infections (ITI-DI) increase the risk of CMV infection. Therefore, we strongly advocate for standardized biopsies in PD-1-related gastrointestinal toxicity patients to identify acute infections at the onset of symptom exacerbation.

## 4 Conclusion

Our case highlights the complexity of irColitis induced by Toripalimab, which is further complicated by CMV infection. Toripalimab proves to be an effective treatment for advanced nasopharyngeal carcinoma, and the irColitis it induces exhibits a strong correlation between symptoms and endoscopic and pathological findings. The diagnosis of CMV infection can be confidently confirmed through standardized biopsy procedures. Our findings may provide valuable insights for the management of acute CMV infection and irAE resulting from Toripalimab treatment in patients with nasopharyngeal carcinoma in the future.

In conclusion, Toripalimab demonstrates satisfactory efficacy in treating advanced nasopharyngeal carcinoma, but it can still lead to irAEs. The endoscopic and pathological findings of PD-1-related colitis correlate with symptoms but are not definitive for diagnosis. Standardized biopsies are recommended to identify acute infections in PD-1-related gastrointestinal toxicity. Considering the risk of CMV infection, vigilance is crucial.

## Data availability statement

The original contributions presented in the study are included in the article/supplementary material. Further inquiries can be directed to the corresponding author.

## Ethics statement

The studies involving humans were approved by Department of Gastroenterology, The Sixth Affiliated Hospital, School of Medicine, South China University of Technology. The studies were conducted in accordance with the local legislation and institutional requirements. The participants provided their written informed consent to participate in this study. Written informed consent was obtained from the individual(s) for the publication of any potentially identifiable images or data included in this article. Written informed consent was obtained from the participant/patient(s) for the publication of this case report.

## Author contributions

CL: Conceptualization, Data curation, Formal Analysis, Writing – original draft. HC: Conceptualization, Data curation, Writing – original draft. HW: Conceptualization, Data curation, Formal Analysis, Writing – original draft. YL: Methodology, Supervision, Writing – original draft. GL: Methodology, Supervision, Writing – original draft. WL: Conceptualization, Funding acquisition, Investigation, Writing – review & editing.
